# Altered Platelets’ morphological parameters in children with type 1 diabetes – a case-control study

**DOI:** 10.1186/s12902-015-0011-8

**Published:** 2015-04-03

**Authors:** Beata Malachowska, Bartlomiej Tomasik, Agnieszka Szadkowska, Anna Baranowska-Jazwiecka, Olga Wegner, Wojciech Mlynarski, Wojciech Fendler

**Affiliations:** Department of Paediatrics, Oncology, Haematology and Diabetology, Medical University of Lodz, 36/50 Sporna St., 91-738 Lodz, Poland

**Keywords:** Platelets, Mean platelet volume, Type 1 diabetes, Children

## Abstract

**Background:**

Platelet hyperreactivity is a factor which contributes towards increased risk of cardiovascular events in adults with type 2 diabetes (T2DM). However, little is known about platelets’ disturbances among children with type 1 diabetes (T1DM). The aim of the study was to investigate whether platelets’ morphology or function are altered in children with type 1 diabetes, potentially predisposing them to cardiovascular events in the future.

**Methods:**

The study group consisted of 389 children with T1DM during the 2008–2010 period. Patients with acute diabetes complications and ongoing infections were excluded from the study. An equinumerous (N = 389), age and sex-matched control group was assembled from children undergoing routine, minor surgical procedures in the same hospital. Platelet: count (PLT), mean volume (MPV), distribution width (PDW) and platelet large cell ratio (P-LCR) as well as HbA1c levels were measured. For statistical analysis we used Chi-square tests, the student’s t-test, one-way analysis of variance (ANOVA), the Pearson’s correlation coefficient and linear regression models in order to adjust for covariates.

**Results:**

MPV, PDW and P-LCR were significantly higher among children with diabetes in comparison with the control group (MPV 10.47+/−0.85 fL vs 10.23+/−0.94 fL, p = 0.0007; PDW 12.09+/−1.80% vs 11.66+/−1.90%, p = 0.0032; P-LCR 28.21+/−6.15% vs 26.29+/−6.38%, p < 0.0001). PLT however, were shown to be similar (263.55+/−60.04 vs 268.77+/−65.78 10^3^/μl; p = 0.5637). In both cases and controls age was inversely correlated with platelet count (for study group: r = −0.30, p < 0.0001; for control group: r = −0.34, p < 0.0001), positively correlated with MPVs (r = 0.20, p < 0.0001; r = 0.26, p < 0.0001), PDW (r = 0.25, p < 0.0001 and r = 0.24, p < 0.0001) and P-LCR (r = 0.26, p < 0.0001; r = 0.26, p < 0.0001). After adjustment for confounding factors, higher platelet counts were associated with poorer metabolic control (beta = 0.20; 0.0001).

**Conclusions:**

Platelets of paediatric patients with T1DM show morphological evidence of hyperreactivity (higher MPV, PDW and P-LCR), while poorer metabolic control increases their number potentially predisposing the patients to future cardiovascular events.

## Background

Type 1 diabetes mellitus (T1DM) is the most common metabolic disease among children, adolescents and young adults and its incidence rate is still rapidly increasing [1]. As the onset of the disease occurs in early life, the afflicted are at great risk of developing cardiovascular disease as a complication of diabetes. Those long-term complications are the leading cause of premature mortality in this group of patients [2,3]. The majority of ischemic events - occur due to intravascular thrombosis. This is a state in which subtle equilibrium between pro- and antithrombotic mechanisms is disrupted and the balance is shifted to favour platelet aggregation and adhesion [4]. The main abnormality observed in diabetic platelets is their hypersensitivity to agonists which leads to their hyperreactivity. It has been shown that platelets obtained from patients suffering from diabetes expressed augmented adhesiveness and aggregation both spontaneous and in response to stimulating agents [5]. Currently available reports have shown unequivocally that increased platelet activity is associated with an elevated frequency of vascular complications in adult patients with type 2 diabetes (T2DM) [4,6]. Based on these observations large clinical trials were undertaken and resulted in the introduction of the antiplatelet drugs as a form of primary prevention in those patients [4].

Mean platelet volume (MPV) is considered as a marker of platelet function. This is based on the fact that the larger platelets are younger, contain more dense granules and thus produce more thromboxane A_2_ [7]. *In vitro* studies showed that increased MPV was associated with greater aggregation in response to ADP and collagen [8]. Larger platelets are more sensitive to platelets’ stimulants are thus are more rapidly recruited to thrombus formation [9]. Taking into consideration aforementioned facts platelet size is thought to be indirect indicator of platelet activity and thus an important factor in micro- and macrovascular diabetes complications [10]. Higher MPV was noted in people with both T1DM and T2DM and these alterations are connected with metabolic control [10,11]. However the influence of metabolic control on platelet morphologic parameters, especially in patients with T1DM, is still unclear and several studies have yielded ambiguous data [12,13]. Studies concerning the link between platelet morphology or function alteration and diabetes were most often conducted on adult populations with T2DM. Our study was aimed to compare morphological platelet parameters – as the indirect indicators of platelet activity - between children with T1DM and their healthy peers. Additionally, we wanted to correlated platelet morphology parameters with glycated haemoglobin level.

## Methods

### Study group

The study group were children with T1DM treated with insulin in the Department of Paediatrics, Oncology, Haematology and Diabetology between years 2008–2010. The study was approved by the institutional Ethics Committee of the Medical University of Lodz and carried out in compliance with the Helsinki Declaration. Written parental consent for participation of their children in the study was obtained. All patients were treated intensively with multiple daily insulin injections (MDI) or continuous subcutaneous insulin infusions (CSII). Criteria for inclusion were set as: complete blood count and HbA1c measurement performed on the same day, duration of diabetes longer than 6 months and age lower than 18 years. Patients with: known monogenic diabetes, acute complications of diabetes (severe hypoglycaemia, diabetic ketoacidosis – defined according to International Society of Pediatric and Adolescent Diabetes Guidelines 2014 [14,15]), ongoing infectious disease or children treated with lipid-lowering or non-steroid anti-inflammatory agents were excluded from the study group. Acute infection in the control group was ruled out on the basis on elevated CRP protein (>5 mg/L) results or elevated leukocyte count (>11.000/μl). Patients with PLT (platelet counts) higher than 500 or lower than 100 (10^3^/μl) were excluded from the study. In the study group acute complications of diabetes or infection were excluded on the basis of medical records from our Department.

### Control group

The control group constituted of non-diabetic, healthy children with blood count results who underwent minor, planned surgical procedures in our hospital in 2008–2010. Patients with infection or comorbidities, which may affect significantly complete blood count parameters, were excluded from the study. The control group was age- and sex-matched to the study group.

### Data collection

Platelets morphology parameters e.g. platelet count (PLT), mean platelet volume (MPV), platelet distribution width (PDW) and platelet large cell ratio (P-LCR) were extracted from routinely performed complete blood count results. The blood counts were performed with *Pentra XLR (Horiba ABX Ltd., Warsaw, Poland)*.

HbA1c measurements were performed with the VARIANT device (Bio-Rad Laboratories, Hercules, CA, USA). Results of HbA1c measurements were in line with NGSP (National Glycohaemoglobin Standardization Program) guidelines on HbA1c measurement standardization as meeting the DCCT standard (http://www.ngsp.org/docs/methods.pdf).

### Statistical analysis

Nominal variables were given as numbers with appropriate percentage whereas continuous variables were presented as means with standard deviation. Chi-square tests were used to test associations between categorical variables. For pairwise comparisons of continuous variables, the student’s t-test was used. For multi-group comparisons one-way analysis of variance (ANOVA) was used. Post-hoc between group comparison were performed with Tukey’s test. Correlations were assessed using the Pearson’s correlation coefficient. Multivariate analyses were performed using linear regression models in order to adjust for covariates. P values lower <0.05 was considered as statistically significant. The sample size was planned using power analysis methods to allow us to detect differences between groups greater than 0.2 of one standard deviation with statistical power of 0.8. This estimation suggested equinumerous groups of at least 384 individuals. Statistical analysis was performed with usage of STATISTICA 10.0 software (Statsoft, Tulsa, OK, USA).

## Results

**Figure 1:**
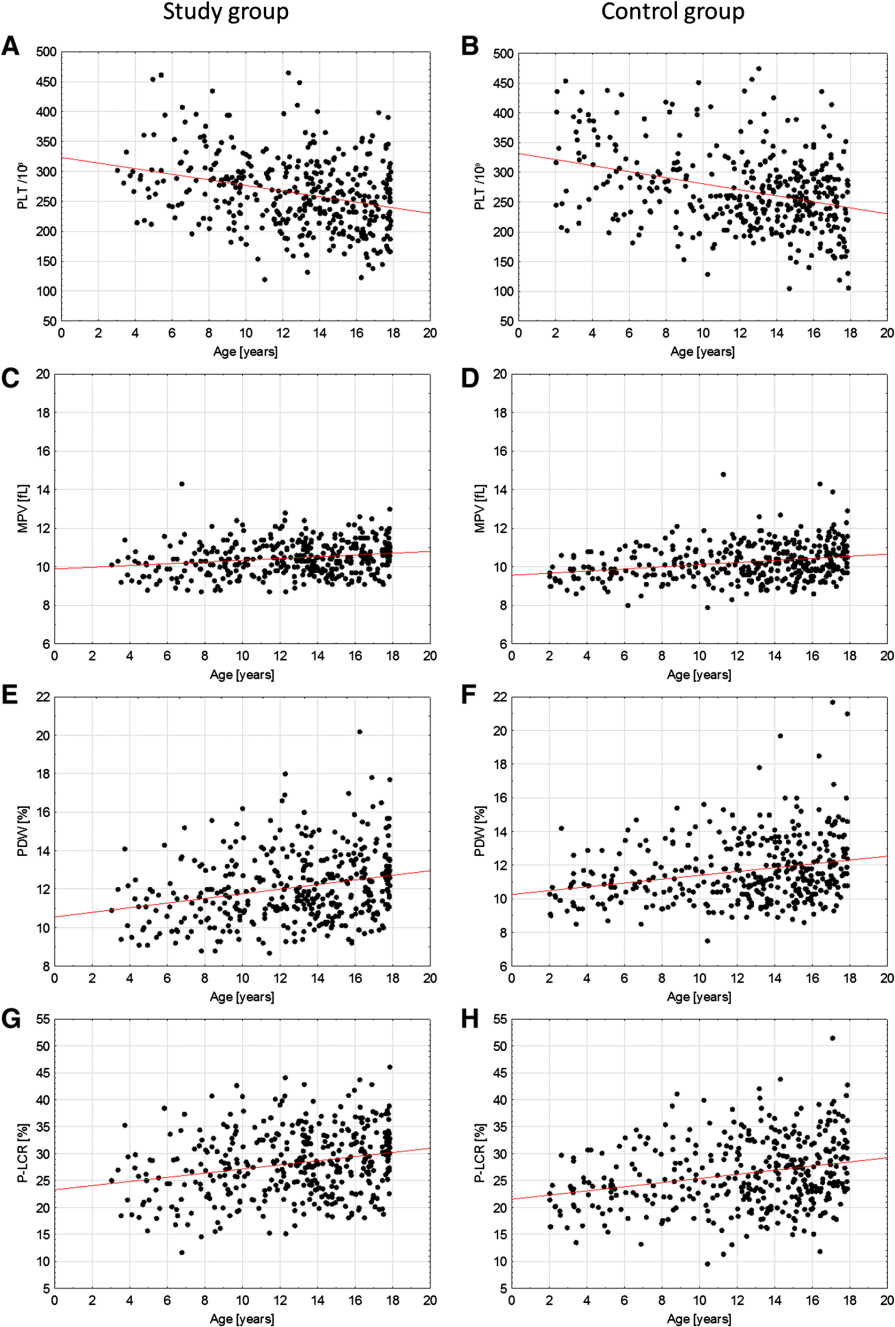
**Correlations of platelet parameters with age in the study and control groups: A/B – Platelet count; C/D – mean platelet volume; E/F – platelet distribution width; G/H – platelet large cell ratio.**

Baseline characteristics of the study groups were shown in Table 1. The study and control groups did not differ in terms of age and sex. In both groups age was negatively correlated with patient’s platelet counts (for study group: r = −0.30, p < 0.0001; for control group: r = −0.34, p < 0.0001), positively correlated with MPVs (r = 0.20, p < 0.0001; r = 0.26, p < 0.0001), PDW (r = 0.25, p < 0.0001 and r = 0.24, p < 0.0001) and with P-LCR (r = 0.26, p < 0.0001; r = 0.26, p < 0.0001) (Figure 1).

**Table 1:** **Clinical characteristics of the studied and control groups**

	**Study group**	**Control group**	**p value**
**Boys/All (%)**	222/389 (57.07%)	207/389 (53.21%)	0.2794
**Age [years]**	12.76+/−3.81	12.33+/−4.34	0.1467
**Treatment type [CSII/all (%)]**	231/389 (59.38%)	---------------	NA
**Diabetes duration [years]**	5.03+/−355	---------------	NA
**HbA1c [%]/[mmol/mol]**	7.54+/−1.49/58.9+/−11.64	---------------	NA

Values are presented as numbers with percentages or means with standard deviations. CSII – continuous subcutaneous insulin infusion, HbA1c – glycated haemoglobin, NA – not applicable.

### Comparison of the study and control group

**Figure 2:**
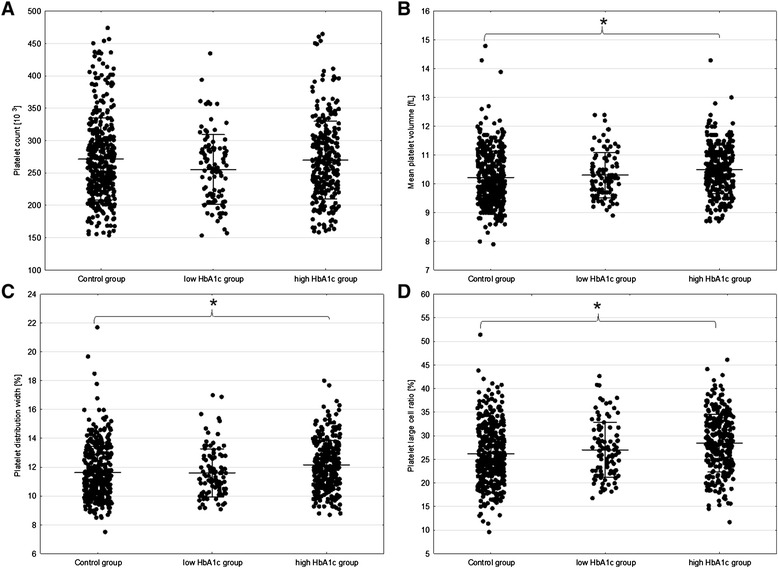
**Comparison of platelets morphological parameters between low (≤6.5%) and high (>6.5%) HbA1c group and control group.** Horizontal lines represent means with standard deviation: **A** – Platelet count (p = 0.0563); **B** – mean platelet volume (p = 0.0004); **C** – platelet distribution width (p = 0.0002); **D** – platelet large cell ratio (p < 0.0001). For post-hoc analyses significant results were indicated with stars.

Mean platelet volume was significantly higher among children with diabetes in comparison with the control group (MPV 10.47+/−0.85 fL vs. 10.23+/−0.94 fL, p = 0.0007). Additionally, platelet distribution width was higher in the in study group than in controls (PDW 12.09+/−1.80% vs. 11.66+/−1.90%, p = 0.0032). This could be associated with greater population of the larger platelets in diabetic children, which were more numerous in the study group (PLC-R 28.21+/−6.15% vs. 26.29+/−6.38%, p < 0.0001). We did not find a significant difference in PLT between study and control group (263.55+/−60.04 10^3^/uL vs. 268.77+/−65.78 10^3^/uL; p = 0.5637) (Table 2). Although the differences became visible when the group with T1DM was divided into well and poorly controlled disease and then compared with control group (below 6.5% of Hb1Ac for good metabolic control according to American Diabetes Association standards 2014 [16]). Surprisingly PLT was the lowest in patients with good metabolic control (255.08+/−53.93 10^3^/uL), PLT was similar in poorly controlled patients and control group (269.84+/−60.08 10^3^/uL vs 271.39+/−63.39 10^3^/uL respectively; p ANOVA = 0.0563). In case of other platelet parameters (MPV, PDW, P-LCR) the lowest values were observed in the control group while the highest in the poorly controlled subgroup of patients with T1DM (Figure 2). This suggested that well-controlled diabetic patients have platelet morphology parameters similar to healthy children.

**Table 2:** **Comparison of platelet parameters between the study groups**

	**Study group**	**Control group**	**p**	**p adjusted for age and sex**
	**Mean+/−Std. Dev.**	**Mean+/−Std. Dev.**		
**MPV [fL]**	10.47+/−0.85	10.23+/−0.94	0.0003	0.0007
**PLT [10** ^**3**^ **/uL]**	263.55+/−60.04	268.77+/−65.78	0.2482	0.5637
**PDW [%]**	12.09+/−1.80	11.66+/−1.90	0.0012	0.0032
**P-LCR [%]**	28.21+/−6.15	26.29+/−6.38	<0.0001	<0.0001

MPV – mean platelet volume; PLT – platelet count; PDW – platelet distribution width; P-LCR – platelet large cell ratio.

### Influence of metabolic control on platelet morphology

To evaluate the influence of HbA1c on platelet parameters we built multivariate regression models to adjust correlations for patient’s age, sex, diabetes duration and diabetes treatment type (Table 3). After adjustment for covariates, only PLT was shown to positively correlate with metabolic control of diabetes (B = 0.20; p < 0.0001). All platelet parameters showed significant correlations with the duration of diabetes. However this effect was due to correlation of diabetes duration with patients’ age.

**Table 3:** **Multivariate regression model results testing for the association of platelet parameters and metabolic control**

	**Diabetes duration**		**HbA1c**	
	**r, p value**	**Adjusted beta, p value**	**r, p value**	**Adjusted beta, p value**
**MPV**	0.15, 0.0026	0.06, 0.2953	0.04, 0.4249	−0.03, 0.5703
**PLT**	−0.11, 0.0230	0.04, 0.4352	0.10, 0.0484	0.20, <0.0001
**PDW**	0.14, 0.0059	0.01, 0.7978	0.07, 0.1929	−0.01, 0.7913
**P-LCR**	0.17, 0.0007	0.06, 0.2726	0.05, 0.2940	−0.03, 0.5746

The effects shown were adjusted for patients’ age, sex and type of treatment. Poorer metabolic control was significantly associated with higher platelet counts. MPV – mean platelet volume; PLT – platelet count; PDW – platelet distribution width; P-LCR – platelet large cell ratio; Beta – partial correlation coefficient.

## Discussion

Our study showed that children with T1DM have elevated platelet volume, wider platelet size distribution width and increased percentage of large platelets. We also found that platelet count was significantly associated with metabolic control. The positive correlation between PLT and HbA1c remained significant after adjustment for confounding factors.

Very little is known about correlation of platelet morphologic parameter with metabolic control of the T1DM in children. A study by Pirgon et al. focused on relation between MPV and HbA1c, but no significant correlation was found [17]. The authors found also that MPV was higher in the group of patients with T1DM in comparison with healthy children, which is in agreement with the results of our study. Both studied populations were in similar age (7.9+/−4.2 years and control group was 7.4+/−3.2 years). The study however was conducted on limited number of patients (N = 56) and the results were not adjusted for patients’ age and sex. In our study MPV in patients with diabetes was only slightly increased (10.47+/−0.85 fL vs. 10.23+/−0.94), but we cannot rule out that this might have clinical implications. Studies conducted on patients with T2DM found significant relationship between micro- and macrovascular complications and MPV [18,19]. This had lead us to hypothesize that even a small increase of MPV might reflect a higher propensity for vascular complications in patients with diabetes than healthy peers. In our study the highest MPV was observed in poorly controlled patients which supports abovementioned hypothesis.

Our recent ongoing study provided information that higher PLT among children with T1DM was connected with shorter time of thrombus formation (unpublished data). Taken into consideration that PLT was found to be independently associated with HbA1c we suppose that poorly controlled T1DM in children have increased platelet activity.

The underlying cause of the observed differences may be related to several factors, most notably platelet function or their turnover. Diabetes mellitus is a prothrombotic state characterized by platelet hyperreactivity, hyperaggregability, enhanced thrombinogenesis and reduced fibrinolysis regardless diabetes type [5]. Platelets in patients with diabetes are more active than in healthy population. Even in the absence of vascular injuries, the platelets have greater expression of the glycoprotein IIb/IIIa receptor, which is the final common pathway of platelet activation. A recent study concluded that advanced glycation end products might be the source of altered platelet function [20]. Thus platelets in patients with diabetes respond to even subthreshold stimuli which leads to faster exhaustion and consumption and, in turn, ends in releasing fresh, hyperreactive platelets [21]. Therefore higher MPV, connected with platelet activity, is often considered as a hallmark of impaired thrombopoiesis in diabetes mellitus [9,22]. It has been shown that platelet population in patients with diabetes exhibits pronounced bimodality i.e. extreme dimensions with an increased number of very large and very small platelets [23]. This was reflected by higher PDW values observed in our patients. The platelet size is also connected with its life time – small platelets are regarded as older, having undergone many episodes of activation whereas large platelets are considered younger and more active [24]. Thus, bimodal distribution of platelet size may be indicative of increased platelet turnover. We observed the highest proportion of large, most active platelets in poorly controlled patients with T1DM.

One could also hypothesise that the observed alterations are the result of primary disorder of platelet precursors, megakaryocytes. Some authors have stated that peripheral platelets are activated in newly diagnosed or even prediabetic patients which could mean that everything is determined at the level of progenitor cells [25]. Many studies report that cytokines such as interleukin-3, 6 and 11 influence megakaryocyte ploidy and may lead to production of more reactive platelets [26,27]. This idea is supported by higher levels of proinflammatory IL-6 in children with T1DM than in healthy peers [28]. Moreover, megakaryocyte functions might be impaired in case of long lasting low glucose levels which are present in well controlled patients and that might result in reduced platelet production. This hypothesis might explain why lower PLT counts were found in the subgroup of patients with low HbA1c levels.

The major clinical question is whether one should counteract increased platelet activity in paediatric patients with T1DM. Maybe good metabolic control in those patients is sufficient to prevent cardiovascular complications and no additional treatment is needed. However recent type reports do not support this hypothesis. In a metaanalysis performed on 2254 patients with 1 diabetes, intensive glycemic control did not affect all-cause mortality or cardiovascular mortality [29]. Nevertheless, we still wait for final answer from DCCT/EDIC which even after 30 years of data collection does not provide a conclusive result [30].

It should be stated that our study has several limitations. Metabolic control was assessed by HbA1c, which corresponds to average glycaemia from the three preceding months. However, platelet lifetime is about 7 to 10 days, which may have affected the observed correlations. One could thus suggest to use a substance such as fructosamine with a shorter half-life time period instead. Unfortunately this marker was not routinely used in our Department throughout the study period. Although we excluded patients with severe infections there is still a possibility that minor infections could affect the platelet parameters. Additionally patients from the control group underwent minor scheduled surgical procedures and the stress conditions connected with this situation might affect the platelet parameters. Another issue which should be taken into consideration in this age group is the matter of pubescence. We countered that by adjusting for patients’ age and sex, but individual levels and potential impact of sex hormone levels on megakaryocytes and platelets are unknown to us.

We believe that metabolic control may be connected with platelet parameters and functions in patients with T1DM. The abovementioned platelet alterations might be associated with diabetes complications particularly because this group of patients is exposed for hyperglycaemia, oxidative stress and proinflammatory state from the early years. The results of our study support the theory that platelet function is altered even in young patients with diabetes potentially contributing to increased cardiovascular risk later on.

## Conclusions

Despite the abovementioned limitations to our knowledge our study is the firstly major report presenting the impact of T1DM on commonly measured platelet parameters in the paediatric population. Further studies are needed as it remains to be evaluated whether antiplatelet drugs may help to safeguard patients with T1DM against future cardiovascular diabetes complications.

## Abbreviations

ANOVA: analysis of variance
Beta: partial correlation coefficient
CRP: C-reactive protein
CSII: continuous subcutaneous insulin infusion
DCCT/EDIC: Diabetes Control and Complications Trial/Epidemiology of Diabetes Interventions and Complications
HbA1c: glycated haemoglobin
MDI: multiple daily injections
MPV: mean platelet volume
NA: not applicable
PLT: platelet count
PDW: platelet distribution width
P-LCR: platelet large cell ratio
